# Diversity of omission responses to visual images across brain-wide regions

**DOI:** 10.1126/sciadv.adv5651

**Published:** 2025-05-21

**Authors:** Noam Nitzan, György Buzsáki

**Affiliations:** ^1^ Neuroscience Institute, New York University, New York, NY, USA.; ^2^ Center for Neural Science, New York University, New York, NY, USA.

## Abstract

An organism’s survival depends on its ability to anticipate forthcoming events and detect discrepancies between the expected and actual sensory inputs. We analyzed data from mice performing a visual go/no-go change-detection task where the sequence of stimulus presentations was intermittently interrupted by omission of a stimulus. The omission of a visual stimulus did not elicit discernable spiking responses in visual cortical neurons. Instead, firing rates between image presentations, including the omission period, ramped linearly and without interruption at the time of the omitted image. Several neuron types in visual cortex neurons were identified with various responses to images and their omissions. A minority of cells in nonvisual areas, including the hippocampus, increased their firing rates at the omitted stimulus onset even when these neurons did not respond to the images. Our study elucidates the origin of omission responses in the visual cortex and sheds light on the role of hippocampal and subcortical circuits in omission detection.

## Teaser

Omission of visual stimuli does not trigger spiking in visual cortical neurons, but certain nonvisual areas respond to omission.

## INTRODUCTION

The brain’s allostatic operations are effective because organisms live in environments with regularities and recurrences, allowing them to predict the future based on past experiences. A fundamental neuronal mechanism in simple and complex brains is detecting deviations from regularity (*1*–*6*). An often-studied experimental paradigm for change detection is the omission of a stimulus embedded among regularly repeating patterns (*7*–*14*). Although no immediate external cue is present, neuronal activity often displays changes at the point in time when a stimulus would have occurred had the sequence continued (*15*). Brain activity during the omission of a scheduled stimulus has been extensively studied in human subjects, and the “responses” have been referred to by various terms, including mismatch negativity, expectation, prediction, novelty, uncertainty, evidence accumulation, decision-making or temporal integration (*16*–*20*), depending on the framework within which the omission paradigm is used.

Two opposite views are discussed to explain the neuronal events in the stimulus omission (SO) paradigm. In the studies of the neurophysiological mechanisms of SO, an early observation is the buildup of a slowly changing potential on the midline scalp, known as contingent negative variation (CNV) or Bereitschaftspotential (*21*, *22*). On a faster scale, SO events induce an earlier negative wave and a later positivity in evoked response recordings from the scalp and magnetoencephalography (MEG) experiments (*23*). Because late (>100 ms) evoked components are often associated with various attentional-cognitive operations (*24*), it has been tacitly assumed that complex circuit operations underlie the detection of SO events. Consequently, SO detection is believed to be relevant to everyday operations, such as speech and music perceptions (*25*–*28*). On the basis mainly of human experiments, and the occasional similarity between omission and stimulus-evoked responses (*14*), it has been suggested that the brain reconstructs a specific neuronal template of the expected but missing stimulus (*29*, *30*). This “internal representation” of the expected stimulus is then compared with bottom-up sensory information to yield a prediction-error signal (*31*).

At the other extreme, the response to SO is viewed as a combination of a temporal expectation and OFF responses to repeated stimuli. This view is supported by the robust SO response in lower vertebrates and simple circuits such as the retina and medullary nuclei (*32*–*34*). A suggested solution for these extreme views is the existence of two types of SO responses, known in humans as “fast” and “slow” (*23*, *35*). The fast type is autonomous, elicited by >4-Hz stimulus trains, and present in reptiles, fish, and mammals. The slow type appears after slower trains (<2 Hz), recorded from the cerebral cortex at a longer latency, requires high-level cognitive processing, and is present only in mammals (*23*). This idea is supported by experiments demonstrating different topographies of the early and late components of scalp-recorded SO responses (*30*).

To relate “simple” and “complex” mechanisms, one needs to record from both cortical and subcortical structures and quantify the nature of SO-induced changes. Because the absence of a scheduled stimulus may temporarily destabilize networks (*2*), a comparison of the stimulus-induced responses before and after the SO may provide further clues about the circuit operations that underlie omission detection. Toward these goals, we analyzed large-scale recordings from multiple visual areas, hippocampus, thalamus, and other subcortical structures made available by the Allen Institute. We find that firing rate changes during SO are ubiquitous and can be observed in both visual and nonvisual areas. However, while firing rate changes in some nonvisual areas, and in particular the hippocampus, were aligned to the expected stimulus onset, neurons in visual areas showed ramping of their firing rates between image presentations but no time-locked responses at the time when the stimulus was expected to occur.

## RESULTS

### Spurious and real modulation of neuronal firing during image omission

**Fig. 1.**
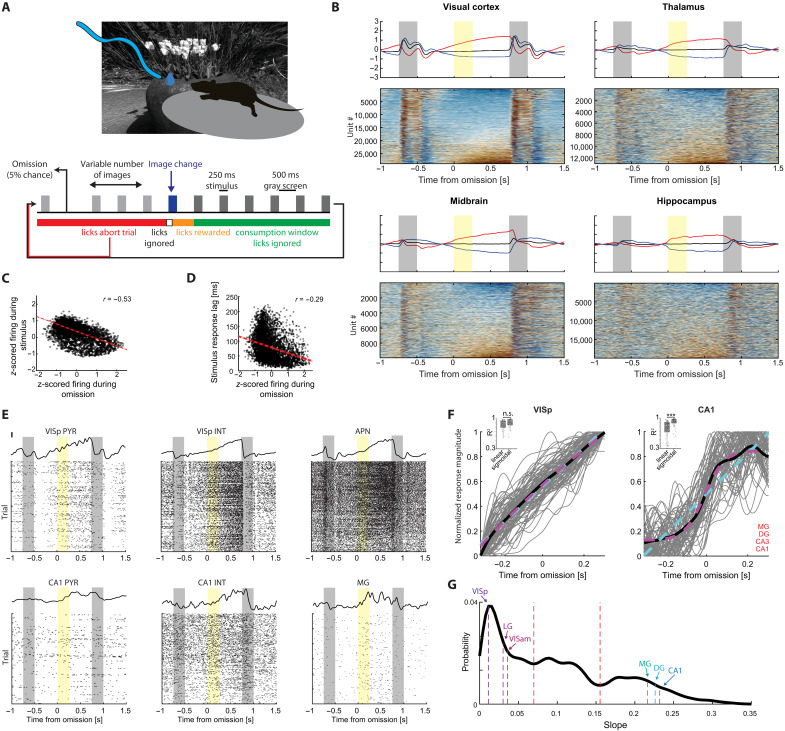
Brain-wide modulation of firing rates during image omission. (**A**) Schematic of the visual go/no-go change detection task. (**B**) Bottom panels: PSTHs showing the responses of cells in each area to the omitted stimulus (time 0), as well as the preceding and following stimuli. Top panels: Average PSTHs for cells that are positively (red), negatively (blue), or not significantly modulated (black) by the omission. (**C**) Firing rate changes during omission of VISp cells, plotted against their firing rates during the stimulus. Red line and shaded area denote regression line and confidence intervals computed using 5000 bootstraps (*n* = 4993 neurons from 96 sessions). (**D**) Firing rate changes during omission of VISp cells, plotted against their stimulus response latency (*n* = 4993 neurons from 96 sessions). (**E**) PSTHs (top) and raster plots (bottom) of six example neurons with a significant firing rate modulation during the omission period. MG, magnocellular nucleus of the thalamus; APN, anterior pretectal nucleus. (**F**) Linear and sigmoidal models were applied to the activity of cells in each area surrounding the omission time. Areas showing ramping activity (VISp is shown as an example, left) were fit similarly well by both models (inset), whereas areas with activity onset around the expected stimulus were better fit by a sigmoidal model (*P* < 0.05, Wilcoxon rank-sum test; CA1 is shown as an example, right). Areas better fit by a sigmoidal model are denoted in the lower right corner of the right panel. Gray, individual sessions; black, average; cyan, linear fit applied to the mean across sessions; magenta, same, for sigmoidal fit. (**G**) Distribution of slopes obtained from sigmoidal fits to population responses during the omission period of neurons in all areas included in the dataset. Troughs in the multimodal distribution (dotted red lines) separate strongly and weakly ramping areas. Medians of example areas are highlighted.

We analyzed data from head-fixed mice trained on a go/no-go visual change detection task (Fig. 1A). Mice were shown a continuous sequence of natural images, each presented for 250 ms. Images were randomly drawn from a set of eight images and a single image was displayed during each trial for a variable number of repetitions (5 to 11 presentations, following a truncated geometric distribution). Subjects received a reward for licking within a 150- to 750-ms window contingent upon the alteration in image identity. Following the task, which lasted 60 min, the exact same sequence of images was replayed but with the water sprout retracted (“passive replay”). Image presentations were stochastically omitted with a 5% probability (Fig. 1A), enabling the investigation of neural firing modulation due to expectation violation. Crucially, image omissions were inserted only between images that remained the same before and after the omission. Following the image omission, the mice reduced their running speed, suggesting they detected the change (fig. S1). As previously reported for the same paradigm (*36*), image omission did not elicit licking responses (fig. S1). Instead, licking rate decreased monotonically between the presented stimuli, without a detectable change in licking rate during image omission. The pupil diameter was also modulated following the omission, yet in contrast to recent human studies (*37*, *38*), image omission induced slight further constriction of the pupil, instead of dilation. While the total luminance of the images and the intermittent gray screen were kept constant, the local luminance of the images was not uniform.

To elucidate the neuronal responses across various brain regions to image omission and compare them with responses to natural images, we computed the peri-stimulus time histogram (PSTH) to the images that immediately preceded and succeeded the omitted images for each neuron in the dataset, averaging across all images (Fig. 1B and fig. S2). PSTHs were sorted by the average *z*-scored firing rate during the omission. This sorting revealed an inverse relationship between the responses of individual neurons to the stimulus and the omitted stimulus. Neurons that were maximally activated or suppressed by the stimulus had decreased or increased firing rates during the omission, respectively. This inverse relationship was consistent across all brain regions included in the dataset (Fig. 1, B and C, and fig. S3). Neurons that increased their activity during the omission period tended to fire early after the presentation of the image, whereas neurons suppressed by the omission exhibited increasing response latencies (Fig. 1D and fig. S3). The firing rates of visual cortical neurons during the omission period were correlated with their spontaneous firing rates, measured at the end of the experiment, while neurons in other brain regions exhibited mixed or weaker correlations (fig. S3). The magnitude of firing-rate increase during the omission was not correlated with the number of stimuli preceding the omitted stimuli within a trial but exhibited a gradual increase throughout the experiment (fig. S4), potentially due to representational drift (*2*). Last, we asked whether omission responses may be stimulus specific, as neural responses to natural images were shown to be highly specific (*2*). We focused on neurons tuned to just one of the eight images and compared their firing rates when the preferred or nonpreferred stimuli were omitted. However, none of the 5802 neurons across all areas included in the dataset showed significantly different responses to omission of their preferred stimulus (*P* > 0.05; fig. S4).

Closer inspection and further analysis of the firing rate changes during image omission showed a complex pattern. In the majority of brain regions, neurons that increased or decreased their rates during the omission window (Fig. 1B, top panel, red and blue, respectively) already began to ramp their firing rates following the offset of the preceding stimulus. Because of the ramping, comparison of firing rates during the omission and the immediately preceding identical epoch showed spurious changes, as if the increased spiking was induced by the image omission. In contrast to the visual cortex, neurons in the hippocampus (DG, CA3, CA1, and SUB) and some nonvisual thalamic nuclei, such as the auditory medial geniculate nucleus (MG), and a few subcortical areas showed sudden changes in their firing rates around the anticipated time of the stimulus and maintained their changed rates until the presentation of the post-omission image (Fig. 1, B and E, and fig. S2). To distinguish ramping and step-like responses, we applied linear and sigmoidal fits to the average responses of up-modulated cells from each region in each session (Fig. 1F). We deemed areas better fit by a sigmoidal function if the distribution of *R*^2^ values was significantly above that of those obtained by a linear fit (Materials and Methods). Visual areas were similarly well described by linear and sigmoidal fits, while some nonvisual areas including the hippocampus (DG, CA1, and CA3; *P* = 0.01, 0.02, and 0.0007, respectively; Wilcoxon rank-sum test) and MG (*P* = 0.02) were better described by sigmoidal fits (Fig. 1F). This was the case also under passive viewing, indicating that the observed firing rate modulation during the omission was not the result of action, motivation, or attention signals (fig. S3).

To further quantify these observations, we plotted the distribution of slopes obtained from sigmoidal fits to all areas (Fig. 1G). This distribution was multimodal, showing three peaks corresponding to linear, intermediate, and step-like responses. All visual cortical areas and the lateral geniculate nucleus were restricted to first mode of the distribution, corresponding to linearly ramping responses, while the majority of hippocampal areas, MG, and a few subcortical nuclei occupied the right tail of the distribution (fig. S3). Commensurate with these findings, visual regions displayed strongest, while hippocampal neurons the weakest ramping of their firing rates across the omission period (*39*) (Materials and Methods and fig. S3). Ramping was steeper when the omitted stimulus was novel (fig. S3), indicating that the magnitude of ramping was governed by the aftereffect of the image response (*2*) rather than omission per se. Consistent with previous observations (*11*, *40*), the average firing rate change was higher among optogenetically identified vasoactive intestinal peptide expressing (VIP) interneurons, compared to both somatostatin expressing (SST) and putative parvalbumin expressing (PV) interneurons, or pyramidal cells (figs. S3 and S5). These analyses indicate that neurons in visual areas display persistent firing patterns long after the image offset (*2*) but are not affected by the absence of a stimulus. In contrast, hippocampal neurons and some subcortical areas, show more robust responses to image omission than to the images.

### Changes in cross-regional interactions during omitted trials

**Fig. 2.**
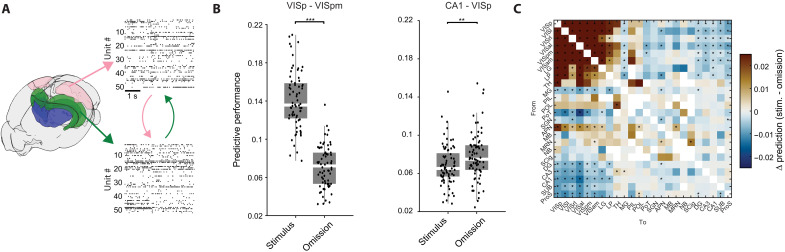
Changes to cross-regional interactions during image omission. (**A**) Graphical depiction of the regression analysis used to assess the strength of interactions between pairs of brain areas. Spiking activity in each area was used to predict activity in a partner area. Raster plots from VISpm (top) and CA1 are shown as examples. (**B**) Left: Distributions of model performance for VISp → VISpm prediction during stimulus and omission. Right: Same, for CA1 → VISp (***P < 0.001 and **P < 0.01, Wilcoxon rank-sum test). (**C**) Average difference in predictive performance for all pairs of areas included in the data. Positive values indicate better prediction during stimulus, negative values indicate better prediction during omission (*P < 0.05, Wilcoxon rank-sum test).

Visual stimuli are known to reduce neural variability (*41*) and increase correlations across visual areas (*42*). Since neuronal firing rates in multiple brain regions undergo changes during the omission period (Fig. 1), we sought to determine whether cross-regional activity is similarly coordinated during the presentation of a visual stimulus and its omission. To address this, we trained a cross-validated ridge-regression linear model (*2*) (Materials and Methods) for each pair of areas in the dataset. This model uses the residual activity (i.e., after subtracting the appropriate PSTH) in one area as its input and outputs the predicted activity in the partner region (Fig. 2A). We constructed separate models for stimulus and omission trials and calculated the interaction strength as the difference in predictive performance between stimulus and omission trials (Fig. 2, B and C). As anticipated, average predictive power between visual areas was higher during visual stimulation. In contrast, nonvisual regions in the hippocampus (DG, CA1, CA3, SUB, and ProS) and nonvisual thalamic nuclei MG and the posterior nucleus of the thalamus (PoT) exhibited stronger correlations with visual areas when the stimulus was omitted, resulting in greater predictive performance (Fig. 2, B and C, and fig. S6). Similar results were obtained using canonical correlation analysis (CCA), which also showed higher correlation between hippocampal and visual areas during omission for the first three canonical dimensions (fig. S6).

### The visual cortex does not encode the identity of the omitted stimulus

**Fig. 3.**
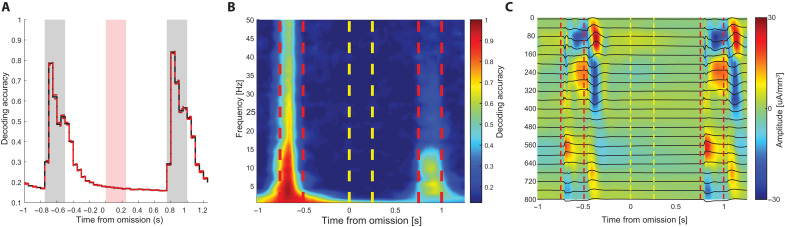
The visual cortex does not encode the identity of the omitted stimulus. (**A**) Average time-resolved prediction accuracy (mean ± SEM) from normalized spike counts in 50-ms windows around the omission time using all visual areas included in the dataset (*n* = 98 sessions). Note the high decoding accuracy for the stimuli preceding and following the omission, but not for the omitted stimulus. Also note that the decoding accuracy remains above chance levels (1/8) even after the stimulus offset (*2*). (**B**) Example stimulus prediction accuracy from the LFP surrounding the omission. Multiple multiclass SVM models were trained to predict the pre-omission stimulus (each for a different frequency band) using all visual cortical areas and layers as features. Each model was then tested on all time points surrounding the omission. Note that the prediction accuracy of post-omission stimulus is above chance levels, while the identity of the omitted stimulus could not be predicted. (**C**) Average CSD profile surrounding the omission from VISp. Note the prominent current sink and sources during the pre-omission and post-omission stimuli, but not during the omission, indicating the lack of substantial inputs during the omission (*n* = 89 sessions).

Spiking responses to omitted stimuli can reflect an unexpected change or reconstruction of the specific omitted image. Previous theoretical work has postulated that unexpected inputs to the cortical network can reinstate the activity of neurons representing the expected stimulus (*31*). To test this hypothesis, we sought to examine whether visual cortical activity encodes the identity of the omitted stimulus. We first trained a multiclass linear decoder to predict the identity of the omitted stimulus, as well as the surrounding stimuli, from the firing rates of individual cortical neurons (Fig. 3A). Decoding accuracy was high during the presentation of the images preceding and following the omission, but not for during the omitted stimulus (Fig. 3A). Similar results were obtained in the hippocampus, although the specificity of image reconstruction was several-fold weaker from both spiking activity and LFP (fig. S7). Because top-down inputs to the visual cortex may result in subthreshold spiking activity, we next examined whether the identity of the omitted stimulus could be read-out from the local field potentials (LFP). To do so, we trained multiple multiclass decoders to predict the identity of the pre-omission stimulus from the power of each frequency between 0.5 and 50 Hz and then tested each decoder on the power of the respective frequency in 100-ms windows surrounding the omission. LFP, especially low-frequency (<15 Hz) activity, was informative about the identity of the presented image, while decoding accuracy during the omission was below chance levels at all frequencies, indicating that the stimulus identity is not coded in the LFP (Fig. 3B). In further support for the lack of involvement of the visual cortex in the detection of the omitted signal, a current source density (CSD) analysis indicated no substantial current sink-sources in VISp during the omission (Fig. 3C).

### Segregation of visual cortical neurons into functional clusters

**Fig. 4.**
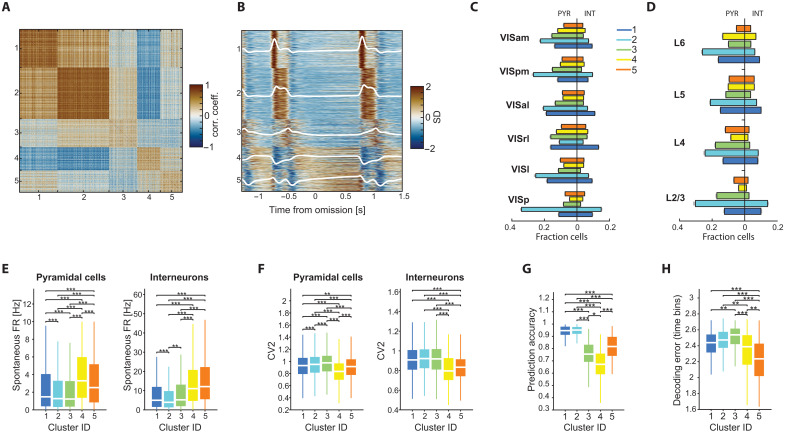
Segregation of visual cortex neurons into functional clusters. (**A**) Correlation matrix showing all visual cortex cells included in the dataset (*n* = 28,926), sorted by cluster identity. (**B**) PSTH of visual cortex cells, surrounding the omission and including the pre- and post-omission stimuli, sorted by cluster identity. The average PSTH of each cluster is overlaid in white. (**C**) Fractions (mean ± SEM) of neurons in each cluster for the different areas, shown separately for pyramidal cells (left) and interneurons (right). (**D**) Same as (C), but for the different cortical layers. (**E**) Distributions of spontaneous firing rates for the different clusters shown separately for pyramidal cells (left) and interneurons (left; *P < 0.05, **P < 0.01, and ***P < 0.001, Kruskal-Wallis with Tukey-Kramer post hoc tests). (**F**) Same as (E), for CV^2^. (**G**) Distributions of stimulus decoding accuracy for the different clusters. (**H**) Distributions of error in decoding the time bin during the omission period from the activity of cells in each cluster.

Our inability to decode the identity of the omitted stimulus from visual cortex activity (Fig. 3), combined with the observation that many visual cortex neurons altered their firing rates even before the expected (omitted) stimulus, suggests that within the current paradigm, omission responses are best explained by a combination of OFF responses and, possibly, temporal expectation signals (*16*, *32*–*34*). This implies the presence of neurons with specific response patterns during the omission period (Fig. 1). To explore this possibility, and to rule out the existence of a minority of visual cortical neurons with specific responses to the omitted stimulus (*10*), we applied *k*-means clustering to the activity of visual cortex neurons during the omission and immediately surrounding stimuli. This analysis yielded five clusters, each with unique stimulus and omission response profiles (Fig. 4, A and B, fig. S8, and the Materials and Methods).

Three of the clusters were up-modulated by the stimulus (STIM-ON clusters), each exhibiting distinct response profiles. Cluster 1 showed a short transient response at the stimulus onset, and in some instances, a smaller response at the offset. Cluster 2 displayed a sustained response that persisted throughout the stimulus duration. Cluster 3 exhibited a gradual increase in firing rate during the stimulus, peaking at the stimulus offset, followed by a decrease during the subsequent omission period. In contrast, clusters 4 and 5 were suppressed during the stimulus presentation (STIM-OFF clusters) and up-modulated during the interstimulus interval (ISI) and omission periods, each with unique response patterns. Cluster 4 displayed step-like OFF/ON activity, whereas cluster 5 gradually increased its activity during the ISI/omission interval, reaching its maximal firing rate at the stimulus onset before rapidly inactivating.

We validated our clustering by training a convolutional neural network (CNN) to predict the cluster labels assigned by the *k*-means clustering (Materials and Methods). To assess whether firing patterns generalize across mice and sessions, the CNN was trained on a randomly selected session and tested on the remaining sessions. Classification accuracy on the test data was well above chance levels, with minimal confusion between clusters (fig. S9).

Responses of visual cortex neurons to natural images are known to exhibit varying degrees of selectivity (*2*). We quantified the variability of responses of the different clusters by comparing their modulation by different images on both recording days (fig. S10). While temporal response profiles across images were similar, the response magnitude of neurons in STIM-ON clusters showed significant differences, indicating stimulus selectivity. In contrast, responses of neurons in STIM-OFF clusters to the different images were not significantly different, indicating a lack of stimulus specificity (fig. S10). Furthermore, similar clustering (>80% overlap) emerged when the omission period was excluded (fig. S8), indicating that these clusters do not result from activity patterns specific to image omission. Last, the temporal dynamic features of the clusters were maintained during epochs of high and low running speed (fig. S8), indicating that while SO is reported by behavior, it did not drive the above-described changes.

All clusters were present across all visual areas, albeit in different proportions: VISp had a higher proportion of cluster 2 neurons, while cluster 1 neurons were most abundant in lateral visual areas (VISl, VISrl, and VISal; Fig. 4C). In contrast, the fractions of clusters 4 and 5 in VISp were lower compared with higher-order visual areas (Fig. 4C). All clusters comprised a mixture of pyramidal cells and putative interneurons (Fig. 4, C and D). Similarly, the relative fraction of neurons in each cluster varied across layers: Cluster 2 was most abundant in superficial layers, while the fractions of neurons in clusters 4 and 5 increased in deeper layers (Fig. 4D). Similar results were obtained when cortical depth was normalized (fig. S8).

To examine whether these cell clusters represent universal classes or are specific to the conditions in which they were classified, we compared them with stimulus-free epochs. Both pyramidal cells and interneurons in clusters 4 and 5 (“stimulus off” clusters) fired at higher rates during a 5-min spontaneous activity epoch compared to neurons in STIM-ON clusters (Fig. 4E). In addition, STIM-OFF cluster neurons fired at more regular intervals, as indicated by their significantly lower CV^2^ (Fig. 4F). Consistent with their higher firing rates during the stimulus, stimulus decoding accuracy was highest for clusters 1 and 2 and lower for clusters 3 to 5 (Fig. 4G). When attempting to decode the exact time bin during the omission period, cluster 5 neurons displayed the lowest decoding error, consistent with previous research showing that ramping neurons can reliably encode elapsed time (Fig. 4H) (*43*).

### STIM-OFF cells are differentially embedded in spontaneous activity patterns

**Fig. 5.**
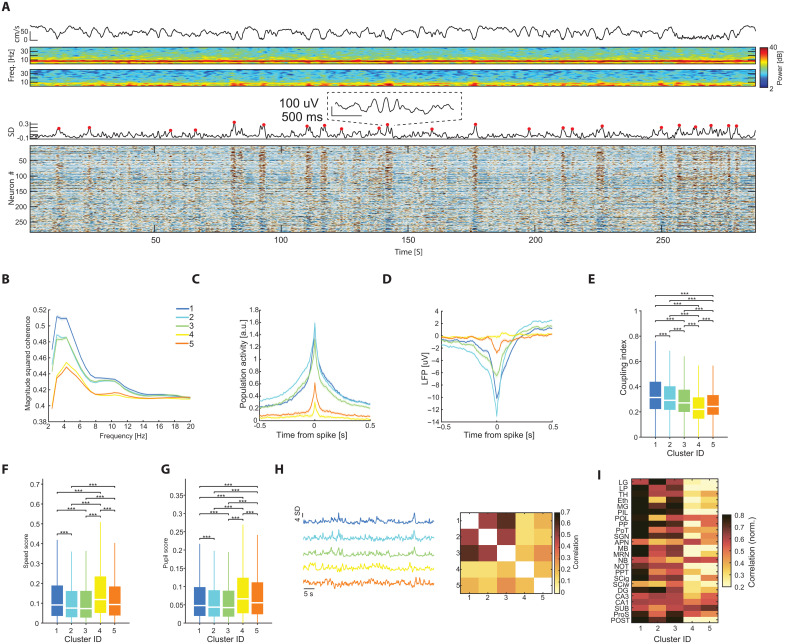
STIM-OFF neurons are differentially embedded during spontaneous activity. (**A**) Top: *z*-scored population firing rate from 5-min spontaneous activity. Population bursts are labeled with red dots. Upper middle: The corresponding spectrogram from a VISp deep layer channel. Lower middle: The corresponding spectrogram from CA1. Bottom: *z*-scored activity of individual visual cortex units, sorted by their omission response in ascending order. Note the cells which are strongly modulated by the omission are suppressed during spontaneous population bursts. (**B**) Magnitude-squared coherence between VISp LFP and visual cortex cells belonging to the different clusters. Note the lower coherence of clusters 4 and 5 in the 3 to 6 Hz band (*n* = 28,926 units in 98 sessions). (**C**) Spike triggered population response (stPR) for the different clusters, averaged across all sessions (*n* = 98 sessions). Note that the omission-modulated clusters (4 and 5) show substantially weaker coupling to the population. (**D**) Same as (C), but for LFP. (**E**) Distributions of population coupling index for the different clusters. Note that clusters 4 and 5 are significantly less modulated by the global visual cortex population activity (***P < 0.001, Kruskal-Wallis with Tukey-Kramer post hoc tests, *n* = 28,926 cells from 98 sessions). (**F**) Distributions of speed scores for the different clusters, calculated from spontaneous activity (***P < 0.001, Kruskal-Wallis with Tukey-Kramer post hoc tests, *n* = 28,926 cells from 98 sessions). (**G**) Same as (F), but for pupil score. (**H**) Left: Example 60 s of spontaneous multi-unit activity summed separately for the different clusters. Note the correlated activity patterns in clusters 1 to 3, but not 4 and 5. Right: Average correlation between visual cortex units belonging to the various clusters, obtained from spontaneous activity. (**I**) Average correlation between visual cortex clusters and population activity in the various subcortical areas included in the dataset. See fig. S11.

It has been shown that cortical neurons exhibit a wide diversity with respect to their coupling to the local population activity, with highly coupled (“choristers”) and uncoupled (“soloists”) cells at the margins of this distribution (*44*). Because population coupling is positively correlated with visually evoked firing rate changes (*44*), we hypothesized that our STIM-OFF clusters will be less strongly coupled to the visual cortex population also in the absence of external inputs. During a 5-min spontaneous activity epoch, mice continued to display active running behavior, as indicated by strong theta-band activity in the hippocampal LFP (Fig. 5A). Occasionally, running was interspersed by brief pauses, which were often accompanied by an increase in the 3 to 6 Hz power in the visual cortex LFP and a transient rhythmic synchrony in the cortical population activity (Fig. 5A). Sorting the activity of individual neurons by their omission modulation score revealed that neurons with high activity during the omission tended to remain inactive, or suppressed their activity during those population bursts. As a result, STIM-OFF clusters showed a weaker coherence to 3- to 6-Hz oscillations in the local LFP (Fig. 5B) and a weaker coupling to the overall visual cortex population (Fig. 5C). Those differences were reflected by a significantly lower coupling index (Materials and Methods), as compared to STIM-ON clusters (Fig. 5E). Similar differences were obtained when examining their coupling to their local LFP (Fig. 5D). STIM-OFF clusters were more strongly modulated by global behavioral indicators, such as running velocity and pupil diameter (Fig. 5, F and G). When separately comparing the correlation of spontaneous activity between the different clusters, we found that STIM-ON clusters were highly correlated among each other, but only weakly correlated with STIM-OFF clusters (Fig. 5H). Similarly, spontaneous correlations between STIM-OFF clusters and multiple subcortical areas, in particular those involved in visual processing, were weaker compared to STIM-ON clusters (Fig. 5I and fig. S11). These results indicate that functionally detected clusters likely correspond to different neuron types, rather than brain state-specific features.

### STIM-OFF cells are less strongly connected within the local population

**Fig. 6.**
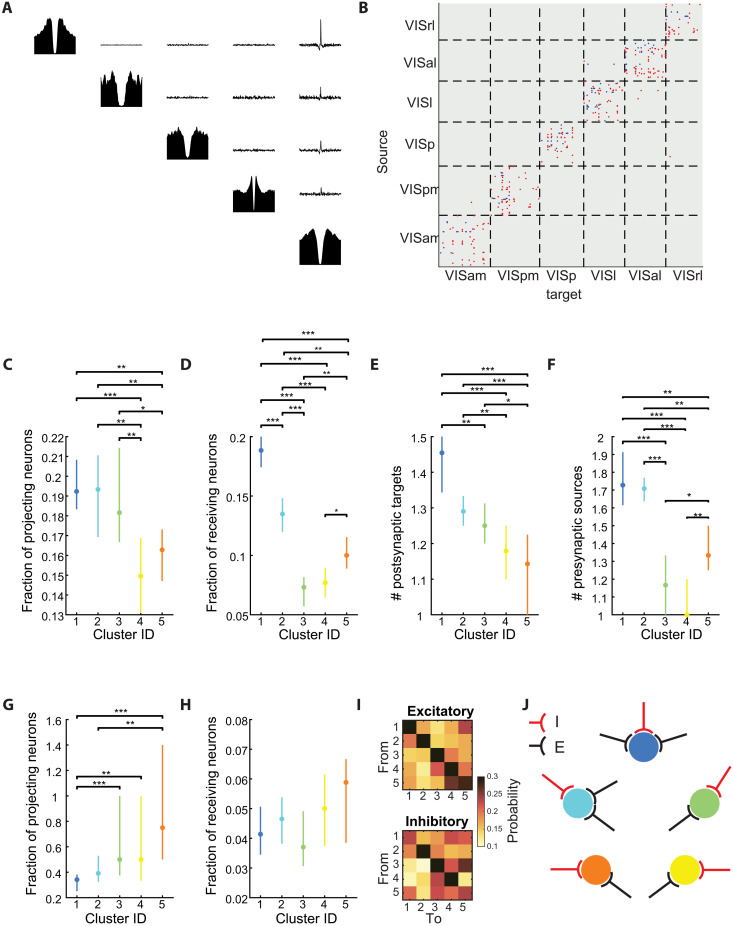
STIM-OFF are less strongly embedded in the local population. (**A**) Example auto- and cross-correlograms (CCGs) between five neurons. Sharp peaks in the CCGs reveal the presence of monosynaptic connections. (**B**) Example connectivity matrix from one session. Red and blue pixels denote excitatory and inhibitory connections, respectively. (**C**) Fractions of neurons with at least one outgoing connection by cluster membership (median ± 95% confidence intervals; *P < 0.05, **P < 0.01, and ***P < 0.001, Kruskal-Wallis with Tukey-Kramer post hoc tests). (**D**) Same as (C), but for incoming connections. (**E**) Average number of postsynaptic targets per session for cells belonging to the different clusters (median ± 95% confidence intervals). (**F**) Same, for but the average number presynaptic sources. (**G** and **H**) Same as (C) and (D) but for inhibitory connections. (**I**) Top: Excitatory connection probability between cells of the different clusters, averaged across sessions. Bottom: Same, but for inhibitory connections. (**J**) Schematic of connectivity imbalance. Black arrows, excitatory inputs; red arrows, inhibitory inputs. Inhibition is equally distributed across the different clusters, whereas clusters 1 and 2 receive significantly more excitatory inputs.

To test the persistent nature of the neuronal clusters, we asked whether the derived classes are differently embedded in the local network. We computed the jitter-corrected cross-correlograms between all pairs of cortical neurons in each session and inferred their monosynaptic connectivity (*45*) (Fig. 6, A and B). As previously reported for the visual cortex (*46*), both putative excitatory and inhibitory connections were mostly restricted to a given visual cortical area, with more limited connections across areas (Fig. 6B). When comparing the fractions of excitatory neurons with at least one outgoing projection in each cluster, we found that STIM-OFF clusters have fewer projecting neurons (Fig. 6C). Those differences were even larger when examining the fractions of neurons that received at least one excitatory connection where neurons in STIM-OFF clusters were 30 to 50% less likely to have detectable excitatory inputs. Cluster 3 neurons showed even lower fractions. Similarly, STIM-OFF excitatory neurons had significantly fewer postsynaptic targets (i.e., lower divergence) and, compared with clusters 1 and 2, clusters 4 and 5 neurons had significantly fewer excitatory targets (Fig. 6, E and F). Yet this was not the case for inhibitory connections, where STIM-OFF clusters had comparable or higher fractions of projecting and receiving neurons (Fig. 6, G, H, and J). When comparing the average connection probability between each pair of clusters, we found that neurons formed both excitatory and inhibitory connections preferably with neurons within the same cluster (Fig. 6I).

## DISCUSSION

We made multiple observations by analyzing large-scale neuronal activity from various brain regions during omission trials in a visual change detection task in mice. In visual areas, the omitted stimulus did not bring about detectable changes at the onset of the omission. Five distinct neuronal response types were identified in visual cortical areas with distinct firing modulation during the stimulus and ISIs, and these clusters remained segregated during the ensuing spontaneous activity. In contrast to visual areas, responses to omitted images in the hippocampus and some nonvisual subcortical nuclei were larger in magnitude than responses to the images themselves, and the elevated/suppressed spiking activity persisted to the presentation of the next image. Those nonvisual areas were better correlated with the visual cortex during SO, as compared to stimulus presentation.

### Spiking activity of neurons during image-omission in visual areas

A genuine omission response should be temporally aligned to the expected timing of the stimulus. The lack of detectable changes of spiking activity in visual cortical neurons at the onset of the SO was an unexpected finding in light of previous studies discussing the physiological responses to expected or missing stimuli (*12*, *13*, *15*, *47*–*49*). Omission responses were hypothesized to stem from multiple, potentially overlapping, processes. On the one hand, omission responses are thought to reflect “off responses,” temporal expectation, and rebound from adaptation, potentially mediated by spike-timing dependent plasticity mechanisms (*33*, *34*, *50*, *51*). This view is supported by the fact that firing rate changes during SO can be observed already in the early stages of the visual pathway such as the retina and even in invertebrates, independently of attention (*23*, *32*). An alternative view is that omission responses reflect a high-level cognitive process where they signal prediction or prediction errors (*13*, *14*, *47*, *52*). This hypothesis is supported by the observation that omission responses scale with the probability of the omitted stimulus (*47*).

In both humans and rodents, omission responses are often reported for auditory and less frequently for visual stimuli (*13*, *23*, *30*, *35*). In human scalp EEG experiments, the omission of an auditory signal produces a signal that resembles the responses produced by the sound itself (*14*, *53*). These observations are corroborated by cellular resolution experiments in rodents, where sound omissions result in a sharp and substantial increase in the firing rates of auditory cortex neurons at the time when it was expected (*15*, *47*, *48*). However, there is evidence suggesting that omission responses may depend on the modality of the omitted stimulus, with various studies reporting substantial differences in the amplitude, latency and morphology of responses to omitted auditory vs. visual stimuli (*13*, *23*, *30*, *35*, *54*). For example, when the final word in spoken sentences was delayed by 600 ms, an emitted potential was detected when the word was expected to occur. In contrast, when words were presented visually, the same manipulation induced a delayed, smaller potential shift (*55*). Those differences were postulated to arise due to a more efficient implementation of predictive mechanisms in the auditory system, potentially evolved in response to the high temporal variability of the auditory stimuli and the fact that, in the auditory system, competing signals overlap, whereas in the visual system, they occlude each other (*13*). One interpretation of the lack of neuronal responses to the omitted image in the visual system is that the mouse visual system is not well equipped to predict fast changes in the visual field, as mice may not experience such sequences in their natural habitat.

To date, a single imaging study in the superficial layers of the primary visual cortex has reported the existence of a small subset (2%) of excitatory neurons showing reliable and rapid calcium responses at the onset of the omitted visual stimulus (*10*). A potential explanation for our inability to detect these neurons is the higher yield of superficial cortical neurons afforded by two-photon imaging. Furthermore, in contrast to the present data, mice in that study navigated in a virtual tunnel and the stimulus was omitted at one location, which remained fixed. As some visual cortex neurons are known to display place cell-like activity (*56*, *57*), it remains to be tested whether those responses signaled prediction error or the subject’s current location/distance traveled (*58*). In addition, the visual flow component intruded by the virtual tunnel may have increased the sensory temporal variability and thus increased the need for predictive processing.

Our findings are consistent with the view that, within the visual cortex, firing rate change during the omission period can be attributed to rebound and temporal expectation signals (*16*, *33*, *34*). This hypothesis suggests competition among distinct neuronal groups and in particular the existence of neurons that are released from inhibition at the offset of the stimulus. This rebound-excited group may then decrease the activity of competing assemblies via lateral inhibition.

In an attempt to identify such neurons in the visual cortex, we used clustering methods on our recorded neuronal populations, revealing five functional subgroups: three with transient, tonic, or gradually increasing excitatory responses, while two showed suppressed activity (*59*). These groups, with distinct distributions in primary and higher-order visual areas and cortical layers, showed stereotypic behavior across mice and persisted whether the clustering was performed with or without the omission window. STIM-ON and STIM-OFF clusters remained segregated during spontaneous activity, indicating their inherent connectivity patterns. Mono-synaptic connectivity analysis revealed marked differences in excitatory and inhibitory connectivity of the different clusters, implying that the functional classification likely corresponded to different neuronal types (*60*). At least a fraction of the ramping STIM-OFF neurons corresponds to VIP interneurons since optogenetically identified superficial VIP neurons show a similar ramping of their spiking activity (*11*, *40*). However, our data suggest that other cell types across different layers, including pyramidal cells, show similar behavior. Consistent with a previous imaging study from superficial excitatory neurons in VISp (*59*), STIM-OFF cells had higher baseline activity and were more strongly modulated by behavioral state variables, potentially reflecting different subcortical modulatory inputs. Even within these functional groups, we failed to identify neurons with reliable spike changes at the onset of SO.

### Neurons in hippocampus and subcortical nuclei respond to omission events

In contrast to visual areas, neurons in hippocampal regions and nonvisual thalamic nuclei, such as MG, displayed a sudden change in their firing rates at the anticipated time of the stimulus. This induced spiking pattern was not image specific, and the step-like change (increase or decrease) of spiking was not affected by the offset of the stimulus. Instead, the step effect in firing rate persisted until the presentation of the next post-omission image presentation (Fig. 1). Areas showing time-locked omission responses were also characterized by stronger interactions with visual areas during image omission compared to stimulus presentations (Fig. 2). This enhanced correlation may be the result of spontaneous activity patterns, which are more effective in driving correlated activity across nonconnected areas (*61*), and might have occurred during the longer ISIs afforded by the image omissions. LFP analysis in either the primary visual cortex or hippocampus showed no sink-source pair during the omission event, demonstrating the absence of time-locked inputs to visual areas from the lateral geniculate nucleus, or from the entorhinal cortex to the hippocampus. This observation reduces or eliminates the sensory thalamus-cortex-hippocampus path as a possible route of neuronal activation in the hippocampus.

Alternative subcortical routes to the hippocampus may involve the nucleus reuniens, the anterior thalamic nuclei, or the medial septum and fimbria/fornix (*62*, *63*). These routes also mediate the startle reflex which is prominently observed in the hippocampus (*64*). The startle reflex is triggered by unexpected stimuli (*65*), and SO may be viewed as an unexpected event. An important computational function of the hippocampus is detecting novelty and monitoring uncertainty by comparing the expected with the unexpected (*1*, *66*). In this role, hippocampal circuits act as a “sentinel,” constantly searching for deviations from predicted outcomes in cortical and subcortical structures (*67*). Hippocampal neurons are known to be modulated by visual inputs (*68*, *69*), and the hippocampus is often depicted at the top of the ventral visual stream (*70*). The observation that hippocampal neurons were modulated by the SO may indicate that deviation from the expected is a fundamental computation to which the navigational system is already evolutionarily primed. Similarly, the prominent omission responses in the auditory MG may indicate that the auditory system is capable of detecting deviation from the expected regardless of the stimulus modality. It is possible that in the present paradigm, the importance of deviation was relatively minor, and the hippocampal output reflecting a discrepancy was not strong enough to recruit the visual cortex.

The step-like changes in firing rates and the persistence of those changes until the next image presentation in subcortical structures indicate that the brain did notice the absence of the scheduled image. However, interpreting the relationship between these firing pattern changes and overt behavior is complex. Monitoring licking responses did not reveal a reliable omission-related response, indicating that the mice distinguished the omission from the change of an image. On the other hand, we found behavioral correlates to the omissions in the mice’s running speed, which slowed down during both active (rewarded) and passive conditions. This behavioral change could have been triggered by the surprise of the omitted image. It is unlikely, though, that the neuronal responses in the hippocampus and other subcortical structures were secondary to speed changes. First, speed was reduced, whereas firing rates increased. Second, firing patterns showed an immediate step-like change after the image omission, whereas speed began to decrease approximately 0.5 s after the missing image. Running speed reached a minimum after the presentation of the post-omission image, by which time the firing rates in all brain structures returned to the regular image-induced patterns.

### Caveats

Rather than suggesting that the visual cortex is not involved in omission detection, the absence of clear omission responses in the visual cortex may be attributed to the experimental design (*71*). In the auditory cortex, many omission-responsive neurons show a significant increase in their firing rates only when the occurrence probability of the omitted stimulus is very high (>75%) (*47*). However, in our change-detection paradigm, image identities changed rapidly, resulting in relatively short sequences of same-identity images. In future experiments, varying the frequency of the omitted stimulus, as well as the ISIs, may reveal important factors involved in omission detection.

Another caveat is the artificial nature of the head-fixed paradigm. During both active and passive conditions, mice retained high running speed (running speed active, 38.7 ± 1.7 cm/s; running speed passive, 42.7 ± 1.9 cm/s; mean ± SEM from 98 mice) even though running was not a requirement for obtaining reward. During the course of the experiment, mice ran on average 3.6 ± 0.16 km, likely associated with excessive calorie loss. This unnatural behavior is likely the result of stress, which could have modulated neuronal activity and obscured omission responses.

## MATERIALS AND METHODS

### Mice

All experiments reported in this paper were performed in accordance with protocols approved by the Allen Institute’s Institutional Animal Care and Use Committee (protocol number 1805). For all analyses presented here, we use data from the Allen Brain Institute Visual Behavior Neuropixels dataset. The dataset included both male and female mice of the following genotypes: 13 wt/wt mice, 29 Sst-IRES-Cre/wt;Ai32(RCL-ChR2(H134R)_EYFP)/wt, and 11 Vip-IRES-Cre/wt;Ai32(RCL-ChR2(H134R)-_EYFP)/wt mice. Forty-eight of these mice were measured across both days.

### Behavior

Mice were trained for 1 hour/day and progressed through multiple training phases by meeting specific progression criteria defined by *d*-prime and the number of contingent (i.e., not aborted) trials per training session. Mice were deemed ready to be transitioned to the recording stage if the following criteria were met during the final training phase: (i) a peak *d*-prime (calculated over a 100 trials rolling window) of >1 for three consecutive sessions; (ii) At least 100 contingent trials on three consecutive sessions; and (iii) mean reward number of >120 over at least three sessions. The active task lasted for 1 hour and consisted of 31.3 ± 0.05 go trials (where the identity of images changed) on day 1 and 29.3 ± 0.07 go trials on day 2 (mean ± SEM).

### Unit quality criteria

For all analyses, we only included units that with less than 0.5% ISI violations, presence ratio higher than 0.9, amplitude cutoff below 0.1, and firing rate higher than 0.1 Hz.

### Cell type classification

Cortical SST and VIP units were identified in the respective transgenic lines based on optogenetic stimulation given at the end of the experiment. The stimulation protocol consisted of 150 10-ms square pulses. Units were considered significantly light modulated if their baseline-normalized firing rate during the stimulation window was at least 1 SD above baseline and if their firing rate distribution during the stimulation window was significantly different from that during the baseline (*P* < 0.05, Kolmogorov-Smirnov test). Units that had a waveform duration shorter than 0.4 ms with a firing rate higher than 10 Hz and which were not SST- or VIP-positive were considered putative PV cells. Units with a waveform duration wider than 0.4 ms and a firing rate lower than 10 Hz that were not SST or VIP positive were considered putative pyramidal cells (fig. S5).

### Event-triggered histograms and firing rate changes during omission trials

PSTHs were computed by counting spiking activity around stimulus time into 10-ms bins. The mean firing rate was then calculated by dividing by the bin size and number of stimuli. The firing rate change was computed as the average *z*-scored activity during the presentation of a stimulus or its omission. Significance of rate change was assessed using a Wilcoxon rank-sum test between the firing rate during the stimulus (omission) and the preceding baseline window.

### Response curve fitting

To characterize regional response profiles during the omission period, we first collected the population responses of all neurons that showed a significant increase in firing rate during the omission period in each session. Population responses during the omission window (±250 ms) were fitted using both a linear univariate regression, as well as using a sigmoidal fit

$$y=\Delta +\frac{A}{1+e^{-\sigma \left(x-c\right)}}$$

Where Δ accounts for vertical offsets, *A* is the amplitude, σ is a slope parameter, and *c* is the shift along the *x* axis. Parameters were optimized by minimizing the sum of squared error. The coefficient of determination (*R*^2^) is reported as the mean on test data (eightfold validation).

### Ramping index

Ramping index (RI) was defined as in (*39*)

$$\text{RI}={\text{log}}_{2}\left(R_{\text{late}}-R_{\text{early}}\right)$$

Where $R_{\text{early}}$ and $R_{\text{late}}$ are the mean responses during the first and last 100 ms of the omitted stimulus, respectively.

### Estimation of pupil area

Eye tracking data were acquired at 30 Hz and preprocessed by the Allen Institute. The pupil diameter, defined as the mean of the pupil height and width, was normalized by the median of each session.

### Pupil and speed scores

The speed and pupil scores for each neuron were calculated as the absolute value of the Pearson’s product-moment correlation between the neuron’s instantaneous firing rate and the mouse’s instantaneous running speed/pupil size.

### CSD analysis

We estimated the CSD using the double spatial derivative in *z* direction. To align CSD profiles from different sessions, we first calculated the CSD in response to a flashing stimulus (fig. S7). In each session, we identified the earliest current sink after stimulus onset, corresponding to the middle layer, and aligned CSD profiles from different sessions using this landmark.

### Clustering of visual cortex units

We used *k*-means clustering to classify visual cortex neurons into different groups. Clustering was performed on the correlation matrix obtained from the omitted stimulus and the immediately preceding and following stimuli (Fig. 4) or on two consecutive stimuli (fig. S8). The optimal number of clusters was determined using two complementary approaches:

1. By calculating the sum of pooled within-cluster pairwise distances dispersion measurement, *W_k_*, for each number of clusters, *k*, and using the elbow method to detect the point where the point slope changes most drastically.

2. Using the gap criterion (*72*), which finds the largest gap value defined as

$${\text{Gap}}_{n}\left(k\right)=E_{n}^{*}\left\{\text{log}W_{k}\right\}-\text{log}\left(W_{k}\right)$$

Where *n* is the sample size. The expected value $E_{n}^{*}\left\{\text{log}W_{k}\right\}$ is determined by Monte Carlo sampling from a reference distribution.

The optimal *k* in each session was computed as the average of these two metrics and the final clustering was repeated using the mode of the optimal k distribution (fig. S8).

### CNN construction

To validate our clustering and to test its generalizability, we used a CNN composed of three convolutional layers, each followed by a max pooling layer and a 2D batch normalization, and one fully connected layer in PyTorch (version 2.2.2). We used a kernel size of 5. Layers contained the following numbers of filters: Layer 1 had 10, layer 2 had 20, and layer 3 had 30. The input to the network was a stacked image of normalized firing rate by trials. Each layers used a standard ReLU function, with the exception of the final output layer. We used a cross-entropy function to minimize the loss. The network was trained on a single session selected in random, after balancing the numbers of neurons in each cluster, and then tested on the remaining sessions.

### Spectral analysis

Spike-LFP coherence was computed using a multi-taper based analysis [Chronux (*73*)] with five tapers and a time-bandwidth product of 3. Spectrograms were constructed using a complex Morlet wavelet convolution with linearly spaced number of cycles.

### Regression models

To predict the time bin during the omission, we used a 10-fold cross-validated linear regression model using the normalized firing rates of neurons from each cluster as input features. Model performance is reported as the distributions of mean absolute error values across folds. To predict target population activity between pairs of brain regions, spiking activity from each area during the 250-ms image presentation/omission window was counted in 50-ms bins, the appropriate PSTH was subtracted from each single-trial response, and the resulting responses were *z* score normalized. Target population activity was predicted using

$${\hat{Y}}_{\text{Ridge}}=XB_{\text{Ridge}}$$

Where $B_{\text{Ridge}}={\left(X^{T}X+\mathrm{\lambda I}\right)}^{-1}X^{T}Y$ is the least-squares solution and λ is a constant that determines the strength of regularization chosen using a 10-fold cross-validation.

### Canonical correlation analysis

We used CCA to assess the strength of interactions between different modes of activity of different pairs of brain regions included in the dataset during stimulus and omission trials. CCA was performed on matrices *X* and *Y* containing concatenated activity from omission trials or stimulus trials with a matched trial number *T* in a pair of brain areas with *n_X_* and *n_Y_* neurons. The results were matrices *A* and *B*, containing *n_X_* and *n_Y_* canonical coefficients, respectively. Sample canonical correlations were computed as the Pearson’s correlation between the projection of each column in *A* and *B* onto *X* and *Y*, respectively.

### Neural correlation analysis

Single-neuron population coupling was computed similar to Okun *et al.* (*44*). In brief, the summed firing rate of *N*-1 visual cortex neurons was binned, zeros centered, and smoothed with a Gaussian kernel of half width 12/$\sqrt{2}$ ms. The coupling index was then computed as the Pearson’s correlation of the left-out neuron’s activity with the summed population activity.

Cross-correlograms were constructed using a bin size of 0.4 ms (Fig. 6) or 10 ms (Fig. 5 and fig. S11). To analyze the coupling between visual cortex clusters and subcortical areas, cross-correlograms were computed using multiunit activity of neurons in each cluster after subsampling to match the number of spikes in each cluster. Cross-correlograms with each area were then scaled with respect to the maximum and minimum cross-correlation rate in each session to allow comparison across sessions. Statistical comparisons between the correlations of different clusters were performed on the area under the cross-correlogram within a ±20-ms window surrounding time zero.

### Decoding of image identity

We used a linear multiclass SVM (MATLAB’s “fitcecoc”) trained using 10-fold cross validation to decode natural image identity. When decoding image identity from the spike times of neurons, input features were min-max–normalized spike counts in 50-ms bins from all units in a given session and brain region. Decoding accuracy is reported as the mean of the cross-validated accuracy.

When decoding image identity from the LFP, visual cortex LFP traces from all layers and areas surrounding the omission and the immediately preceding and following stimuli were filtered between 0.5 and 50 Hz and the power at each frequency was obtained using complex Morlet wavelet convolution. For each frequency, we trained a linear multiclass SVM to predict the identity of the stimulus preceding the omission using the integrated power of that frequency during the stimulus presentation. We then used the trained models to predict the stimulus identity from the integrated power at each frequency during 100-ms windows with a 25-ms sliding step.

### Statistical analyses

Data were analyzed in Matlab (2021b). Throughout the paper, data are presented as mean ± SEM or, when indicated, median ± 95% confidence intervals. Data are displayed as box plots representing median, lower, and upper quartiles and whiskers representing most extreme data points or as median ± 95% confidence intervals computed from 5000 resamples. Statistical tests for two groups were performed using Wilcoxon rank-sum test or signed-rank test when applicable. Statistical tests for multiple groups were performed using Kruskal-Wallis test followed by Tukey-Kramer post hoc tests.
