# Supplementary material for: Diversity of omission responses to visual images across brain-wide regions
Source: Sci Adv. 2025 May 21;11(21):eadv5651. doi: 10.1126/sciadv.adv5651 (PMC12094227; doi:10.1126/sciadv.adv5651)
Supplement: Supplementary file 1 — Figs. S1 to S11 [file sciadv.adv5651_sm.pdf]

Science Advances
AAAS

Supplementary Materials for

Diversity of omission responses to visual images across brain-wide regions

Noam Nitzan and György Buzsáki

Corresponding author: György Buzsáki, gyorgy.buzsaki@nyulangone.org

Sci. Adv. **11**, eadv5651 (2025)
DOI: 10.1126/sciadv.adv5651

This PDF file includes:

Figs. S1 to S11

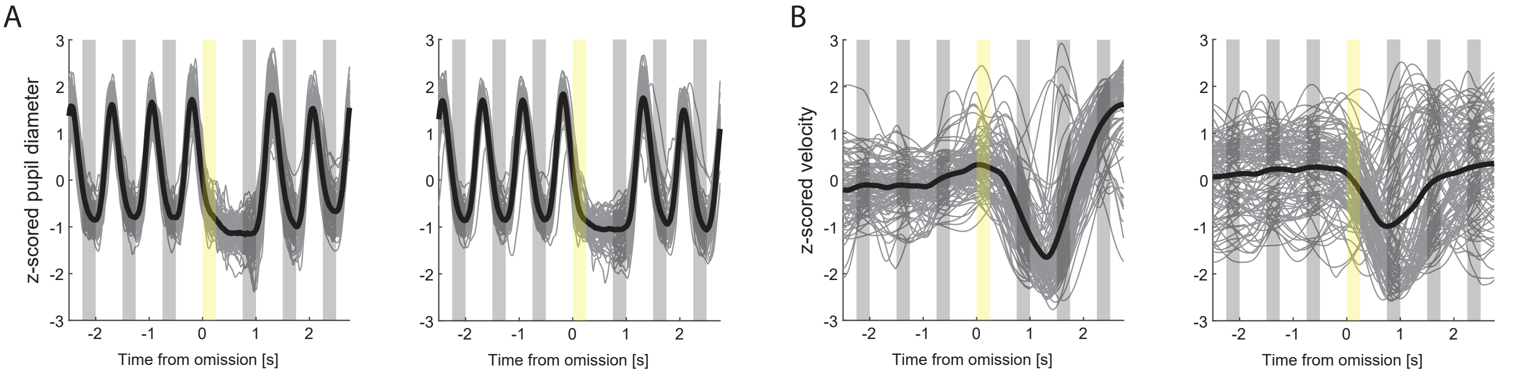

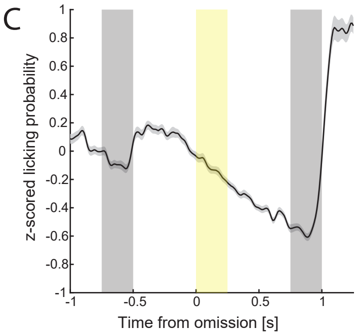

Figure S1. Behavioral changes around stimulus omission. A) Left: Session averages (gray) and grand average (black) of z-score normalized pupil diameter surrounding image omission (yellow shading), as well as the preceding and following stimuli (gray shading) during the active task (n = 98 sessions). Right: Same, for passive viewing conditions. **B)** Same as A, but for running velocity. **C)** Z-scored licking rate surrounding image omission (mean ± SEM, n = 98 sessions). Note that the images preceding and following the omission were never the changed (i.e., rewarded) image. Mice increased their licking rate following images, but not following image omissions.

A

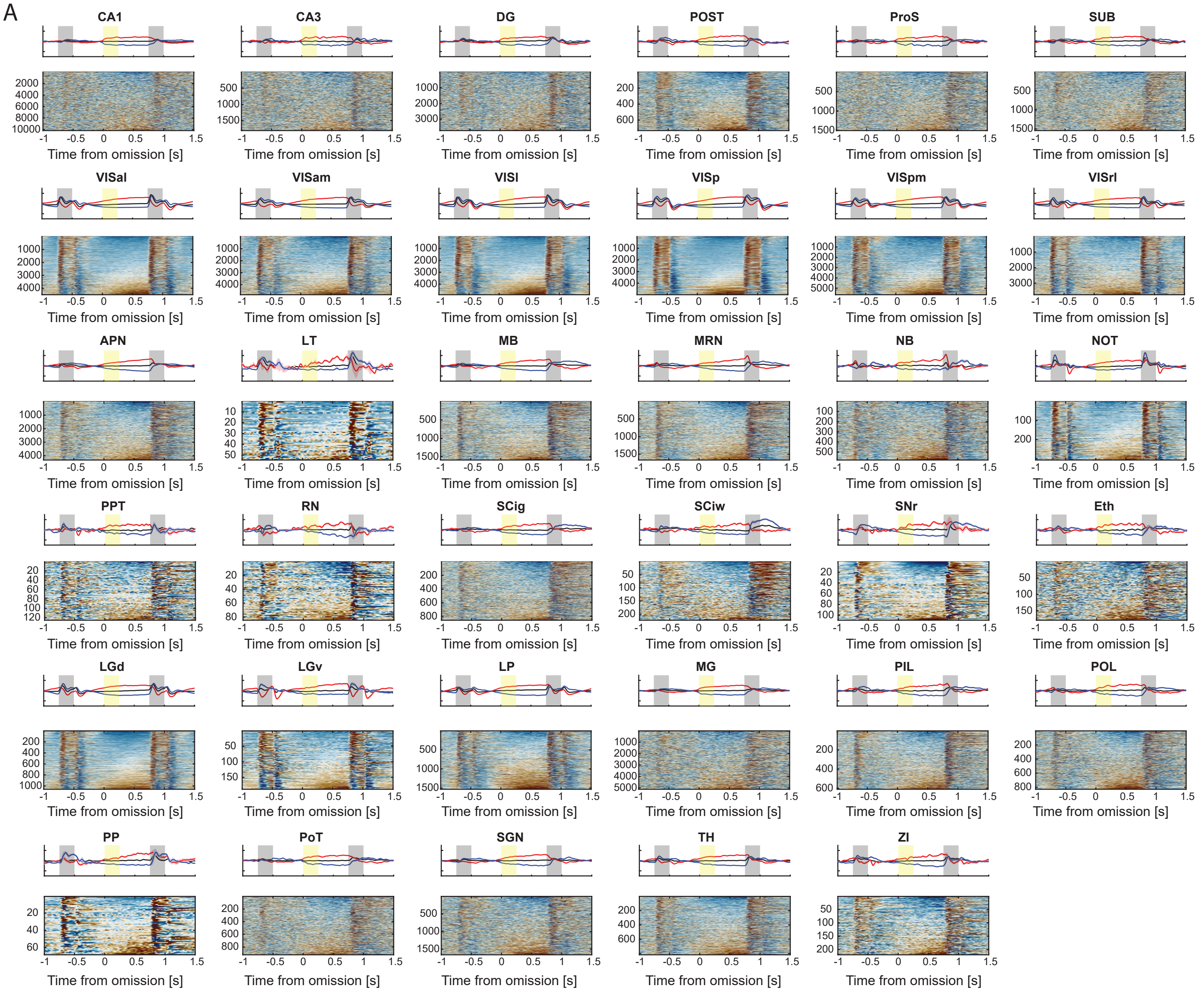

B

Acronym	Full name	# neurons (sessions)	Acronym	Full name	# neurons (sessions)
CA1	Field CA1	10192 (98)	PPT	Posterior pretectal nucleus	125 (17)
CA3	Field CA3	1812 (89)	RN	Red nucleus	84 (15)
DG	Dentate gyrus	3732 (97)	SCig	Superior colliculus, motor related, intermediate gray layer	857 (38)
POST	Postsubiculum	730 (60)	SCiw	Superior colliculus, motor related, intermediate white layer	223 (15)
ProS	Prosubiculum	1501 (49)	SNr	Substantia nigra, reticular part	109 (12)
SUB	Subiculum	1546 (64)	Eth	Ethmoid nucleus of the thalamus	178 (11)
VISal	Anterolateral visual area	4500 (96)	LG	lateral geniculate complex	1248 (51)
VISam	Anteromedial visual area	4836 (94)	LP	Lateral posterior nucleus of the thalamus	1531 (75)
VISl	Lateral visual area	4891 (93)	MG	Medial geniculate complex	5131 (83)
VISp	Primary visual area	4993 (96)	PIL	Posterior intralaminar thalamic nucleus	611 (42)
VISpm	posteromedial visual area	5623 (97)	POL	Posterior limiting nucleus of the thalamus	828 (51)
VISrl	Rostrolateral visual area	3740 (94)	PP	Peripeduncular nucleus	68 (13)
APN	Anterior pretectal nucleus	4315 (87)	PoT	Posterior triangular thalamic nucleus	924 (55)
LT	Lateral terminal nucleus of the accessory optic tract	54 (9)	SGN	Suprageniculate nucleus	1661 (63)
MB	Midbrain, misc. nuclei	1589 (84)	TH	Thalamus, misc. nuclei	819 (72)
MRN	Midbrain reticular nucleus	1665 (69)	ZI	Zona incerta	219 (18)
NB	Nucleus of the brachium of the inferior colliculus	578 (35)			
NOT	Nucleus of the optic tract	307 (46)			

Figure S2. Peri-stimulus and omission responses for individual areas. A) Bottom panels: PSTHs showing the responses of cells in each area to the omitted stimulus (time 0), as well as the preceding and following stimulus for each area included in the dataset. Top panels: Average PSTHs for cells that are positively (red), negatively (blue) or not modulated (black) by the omission. **B)** Acronyms, full names and numbers of neurons and sessions of all areas included in the dataset.

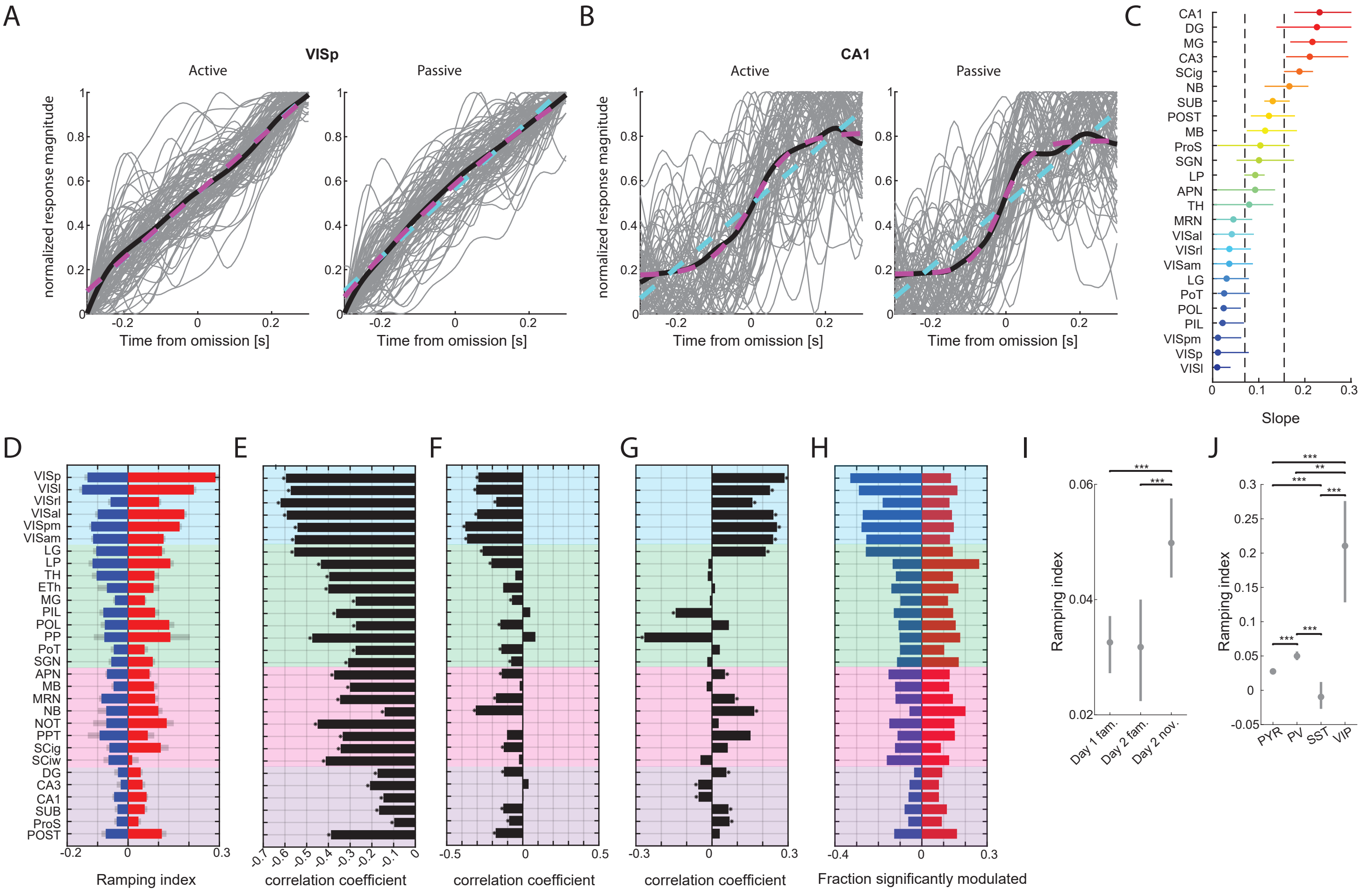

Figure S3. Characterization of omission responses across areas. A) Left: Average normalized activity of VISp neurons from all sessions surrounding omission time during the active part of the task (n=89). Black, average; Cyan and magenta, linear and sigmoidal fits applied to the mean across sessions. Right: Same, for passive viewing. **B)** Same as A, but for CA1 neurons (n=98 sessions). **C)** Slopes (median ± 95% confidence intervals) obtained from sigmoidal fits to the average firing rate of positively-modulated neurons (n ≥ 5 sessions per area; areas with less than 5 sessions are not included). Dashed lines, troughs in the multi-modal distributions (Fig. 1G). **D)** Ramping index (mean ± SEM) for negatively (blue) and positively (red) modulated cells in each area. Note that non-visual areas display weaker ramping compared to visual areas. **E)** Correlation between omission and stimulus modulation for the different areas (*p<0.05) **F)** Average correlation between omission modulation and stimulus response latency of neurons in the different areas (*p<0.05, one-sided t-test). **G)** Correlation between spontaneous firing rates and omission scores of neurons in the different areas (*p<0.05, one-sided t-test). **H)** Fractions of positively (red) and negatively (blue) omission modulated cells in each area. **I)** Ramping index (median ± 95% confidence intervals) in response to omitted stimuli belonging to the different stimulus categories (***p<0.001, Kruskal-Wallis with Tukey-Kramer post-hoc tests, n=30667 neurons from familiar sessions and n=24482 from novel sessions). **J)** Ramping index shown separately for the different cell types (**p<0.01, ***p<0.001, Kruskal-Wallis with Tukey-Kramer post-hoc tests, n_{PYR}=20390,n_{PV}=1430,n_{SST}=634,n_{VIP}=55).

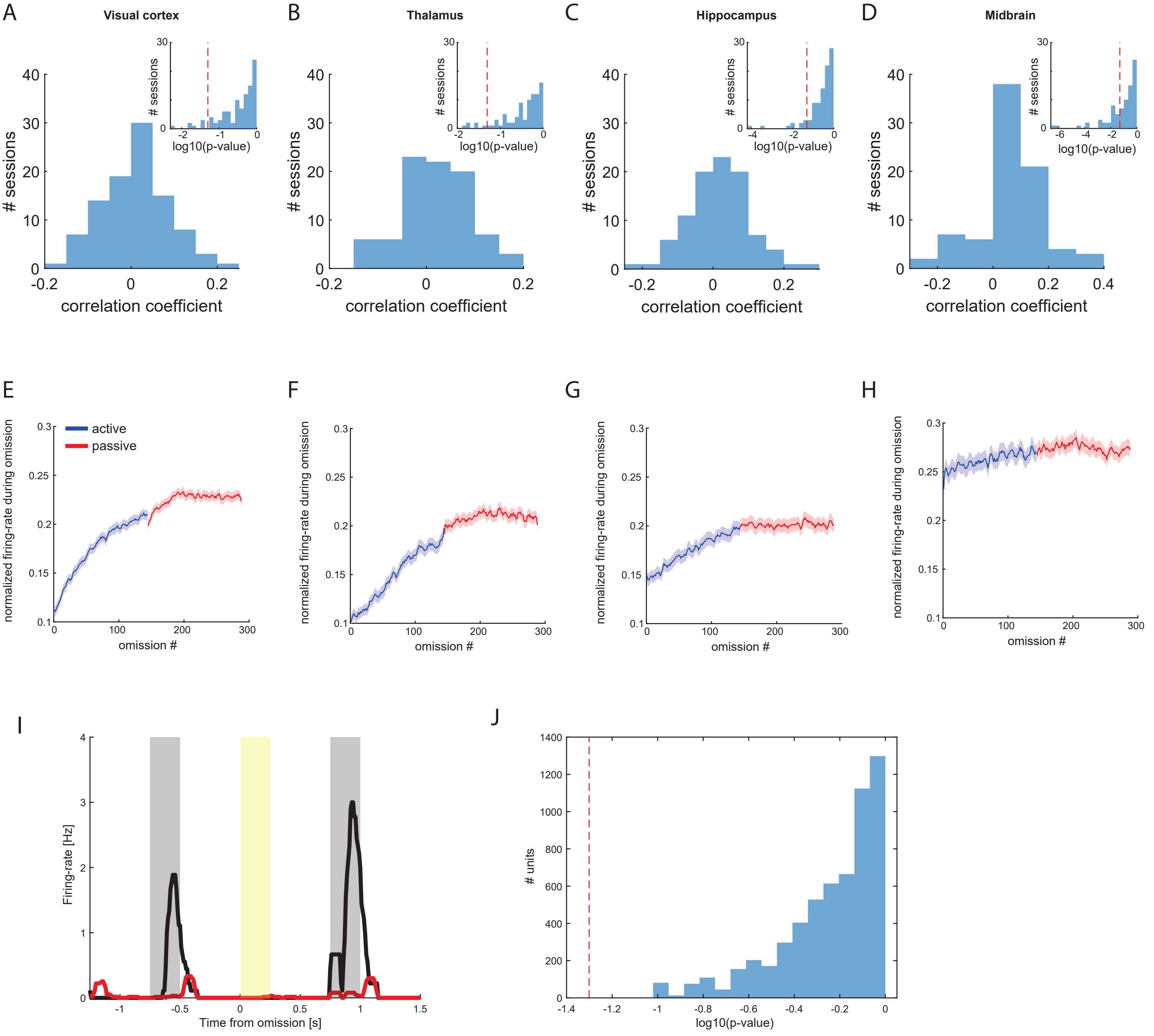

Figure S4. Dynamics and specificity of omission responses. A) The distribution of correlation values between the number of stimuli presented in a trial before the omission and the firing rate of the positively modulated population in the visual cortex across is weak and centered around 0 (0.018 ± 0.007 ,mean ± SEM). Inset: Distribution of p-values obtained from the correlation. Dashed red line, p = 0.05. Note that the vast majority of sessions do not show a significant correlation between the number of stimuli preceding the omission and the populaiton firing during the omission (n = 98 sessions). **B-D)** Same, for thalamus, hippocampus and midbrain, respectively. **E)** Temporal dynamics of population firing (for omission positively-modulated visual cortex neurons) throughout the session (mean ± SEM, n = 98 sessions). Blue, active task; Red, passive replay. **F-H)** Same, for thalamus, hippocampus and midbrain, respectively. **I)** PSTH of an example visual cortex neuron with selective responses to only one of the eight images triggered on omissions of the preferred (black) or non-preferred stimuli (red, averaged across all non-preferred stimuli). **J)** Distribution of p-values obtained from comparing the distributions of firing-rates of all units selective to one image when the preferred stimulus was omitted and when the non-preferred stimuli were omitted. Dashed red line, p=0.05 (n = 5802 units from 98 sessions; Wilcoxon rank-sum test).

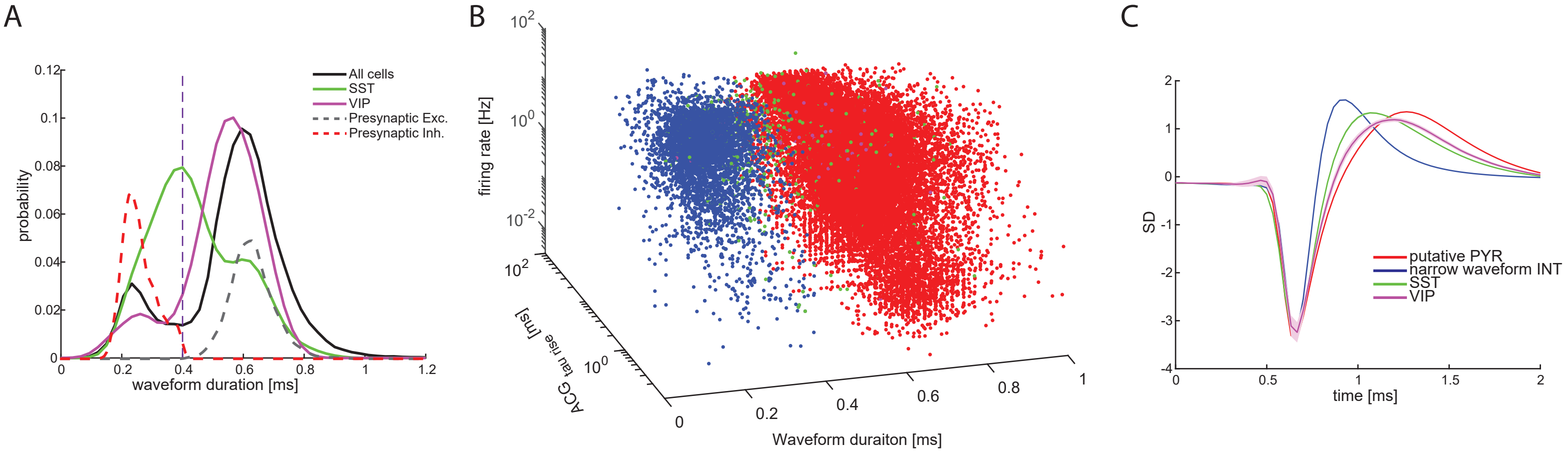

Figure S5. Cell-type classification. A) Probability distribution of all cells included in the dataset (black). Fast-spiking interneurons were classified based on the trough in the bimodal distribution. Distributions of optogenetically identified SST and VIP cells are plotted in green and magenta, respectively. Distributions of presynaptic excitatory and inhibitory cells identified using monosynaptic connectivity analysis, are shown in gray and red dashed lines, respectively. **B)** Scatter plot showing the waveform duration, ACG time constant and firing rates of cells included in the dataset. Putative pyramidal cells are plotted in red, fast-spiking interneurons are shown in blue. SST and VIP cells are plotted in green and magenta, respectively. **C)** Average waveforms (mean ± SEM) of the different cell types.

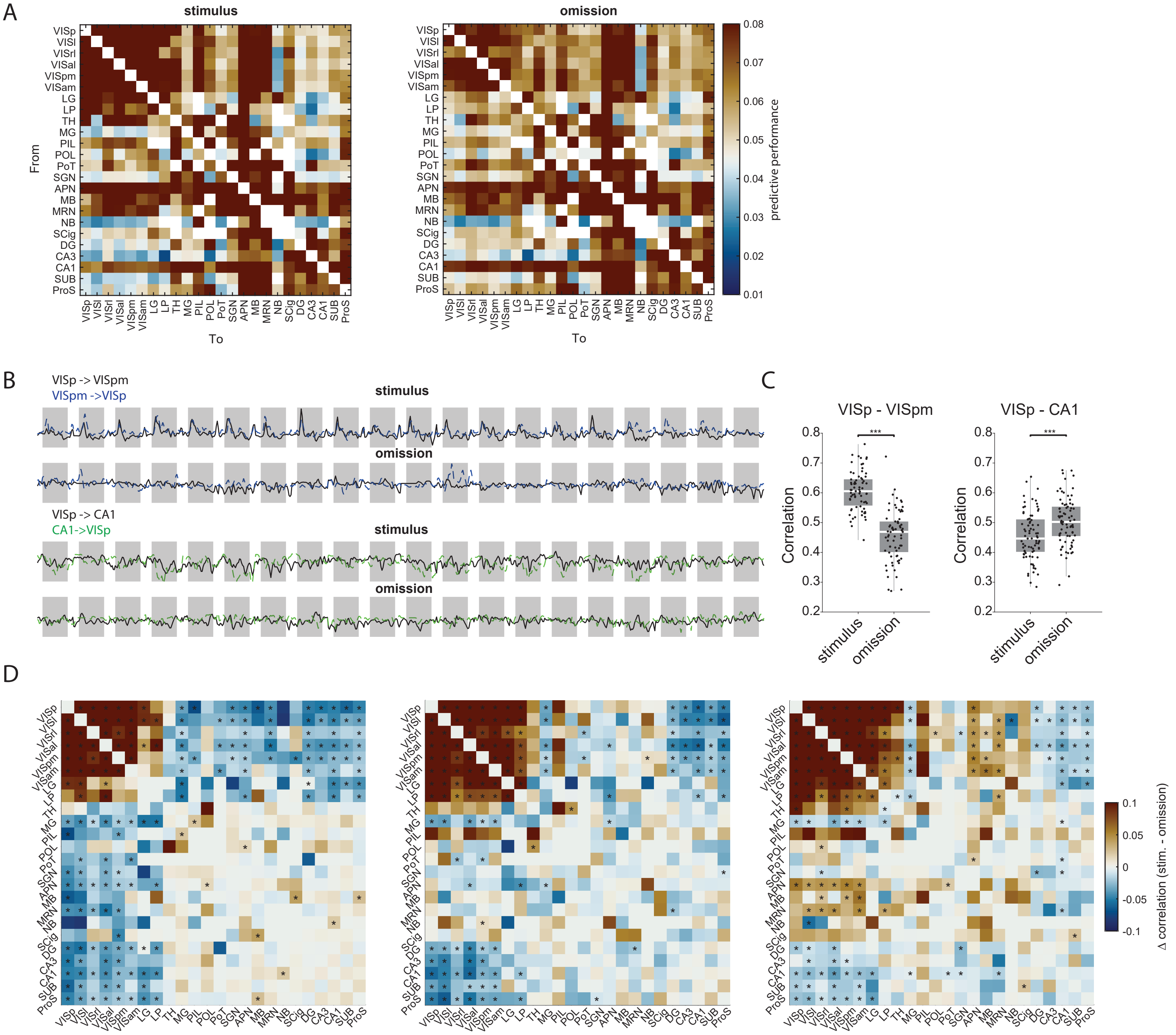

Figure S6. Changes to cross-regional population correlations during image omission. A) Average predictive performance of a ridge-regression model for all pairs of areas included in the data. See Fig. S2 for the numbers of neurons and sessions for each area. **B)** Example projections of the first CCA mode for stimulus and omission trials from one session, shown for 2 pairs of areas. **C)** Left: Distributions of correlation values between VISp and VISpm for the first CCA mode. Right: Same, for VISp and CA1. Note that the correlations between VISp and CA1 are significantly higher during omission (***p<0.001, Wilcoxon rank-sum test). **D)** Matrices showing the difference between correlation obtained from stimulus trials and omission trials between each pair of areas for the first 3 CCA modes. Positive values indicate higher correlation during the stimulus, whereas negative values indicate higher correlation during omission.

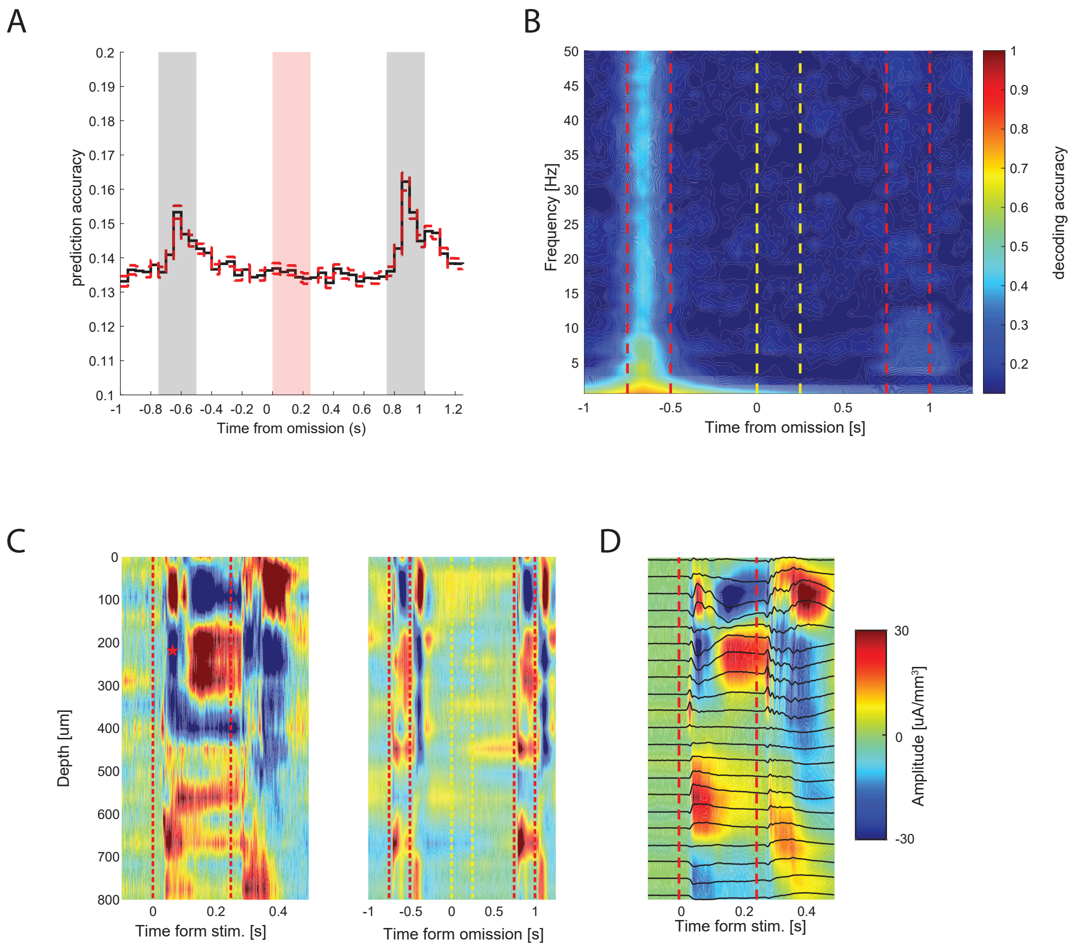

Figure S7. Decoding of omission responses. A) Average time-resolved prediction accuracy (mean ± SEM) from normalized spike counts in 50-ms windows around the omission time using all hippocampal areas included in the dataset (n=98 sessions). **B)** Example stimulus prediction accuracy from the hippocampal LFP surrounding the omission. **C)** Right: Example average CSD of 250-ms flashing stimulus from one session. Red star denotes earliest strongest current sink which was used to align CSD profiles across sessions. Left: Same, but for the omitted stimulus as well as the preceding and following natural images stimuli. **D)** Average flashing stimulus CSD from all sessions (n = 89 sessions where VISp was available).

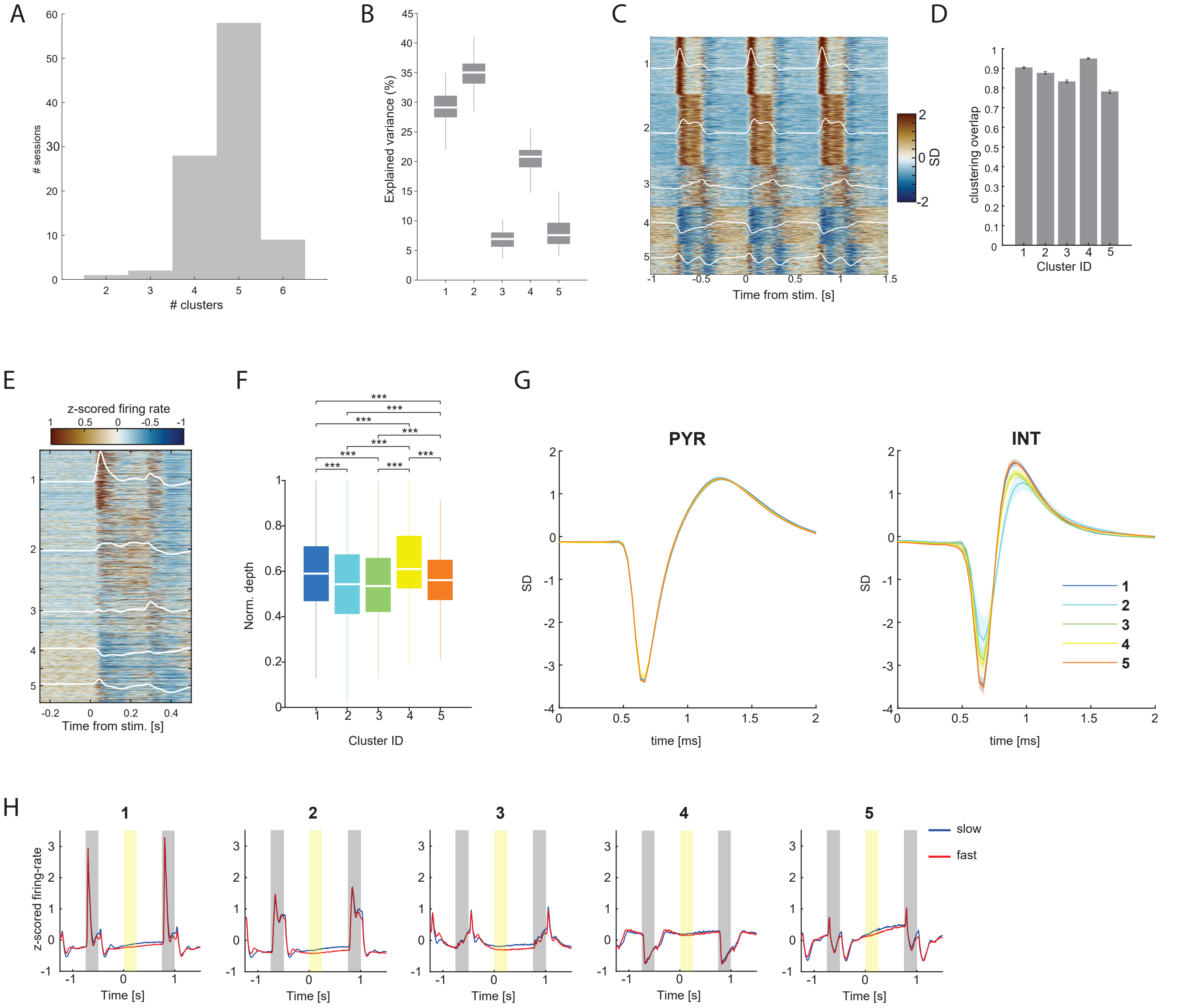

Figure S8. Clustering of visual cortex cells. A) Distribution of the optimal number of clusters using a combination of the elbow method and gap statistics (n = 98 sessions; Methods). **B)** Box plots showing the distributions of variance explained by each cluster's response across sessions (n=98 sessions). **C)** Clustering of visual cortex cells was repeated excluding the omitted stimulus. PSTH of visual cortex cells sorted by cluster identity. The average PSTH of each cluster is overlaid in white. Note the similarity to Fig. 4B. **D)** Fraction of cells (mean ± SEM across sessions) with overlapping clusters using clustering with or without the omission period (n=98 sessions). **E)** PSTH of visual cortex cells, triggered on 250-ms white full field flashes, sorted by cluster membership. The average PSTH of each cluster is overlaid in white. Note the similarity in temporal dynamics with responses to natural images. **F)** Distributions of normalized depth for each cluster. Note that clusters 4-5 are, on average, deeper than clusters 1-3 (***$p<0.001$, Kruskal-Wallis with Tukey-Kramer post-hoc tests). **G)** Average waveforms (mean ± SEM) of pyramidal cells (left) and interneurons (right) in each cluster. **H)** Average omission PSTHs for the different visual cortex clusters, plotted separately for omissions occurring during the lowest (<26.65±1.65 cm/s, mean ± SEM, blue) or highest (>54.75±2.23 cm/s, red) running velocity quartiles. Note that the underlying demporal dynamics during the stimulus and omission are preserved regardless of running speed (n = 6769, 9120, 4985, 4136 and 3916 units in clusters 1-5, respectively).

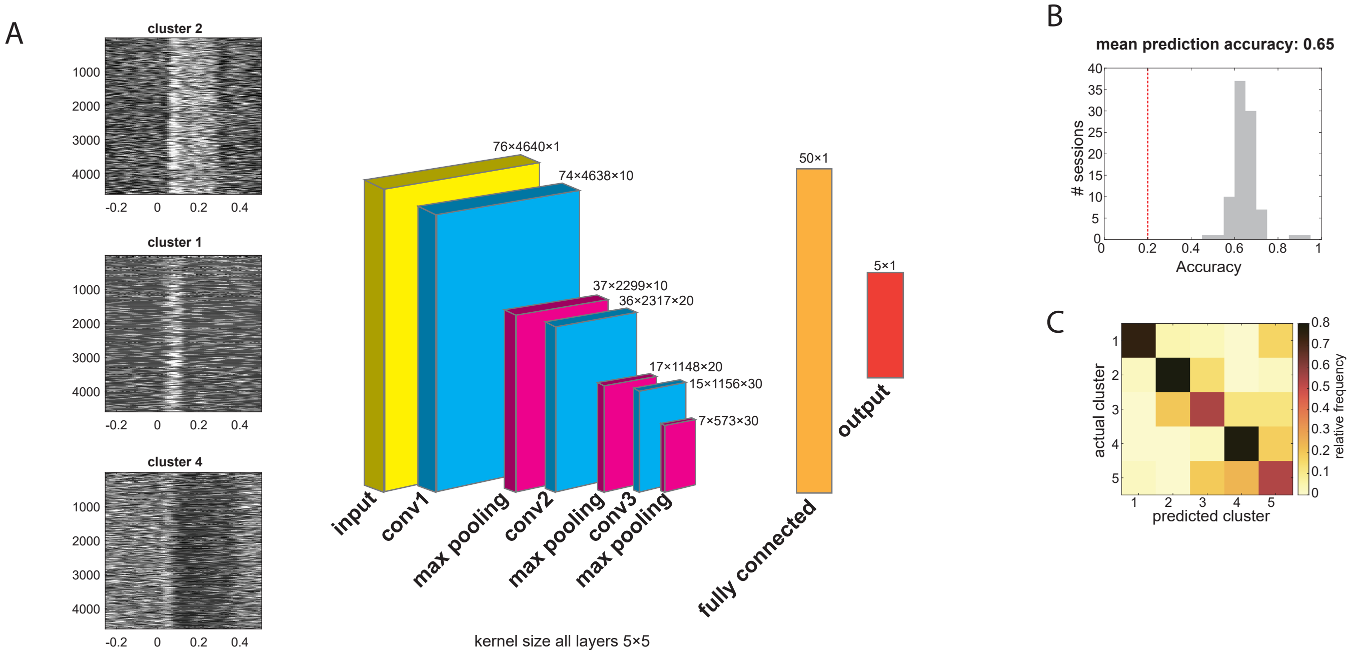

Figure S9. Classification of visual cortex neurons. A) A convolutional neural network was used to predict the labels assigned to visual cortex neurons by k-means analysis. Left: 3 example inputs to the network, presented as a matrix of time by trials, centered around stimulus onset. Right: Inputs were passed through 3 convolutional and max-pooling layers and a fully connected layer, before returning the predicted labels probabilities. **B)** Distribution of classification accuracy, red dashed line, chance levels (1/5). **C)** Average confusion matrix (n=88 sessions).

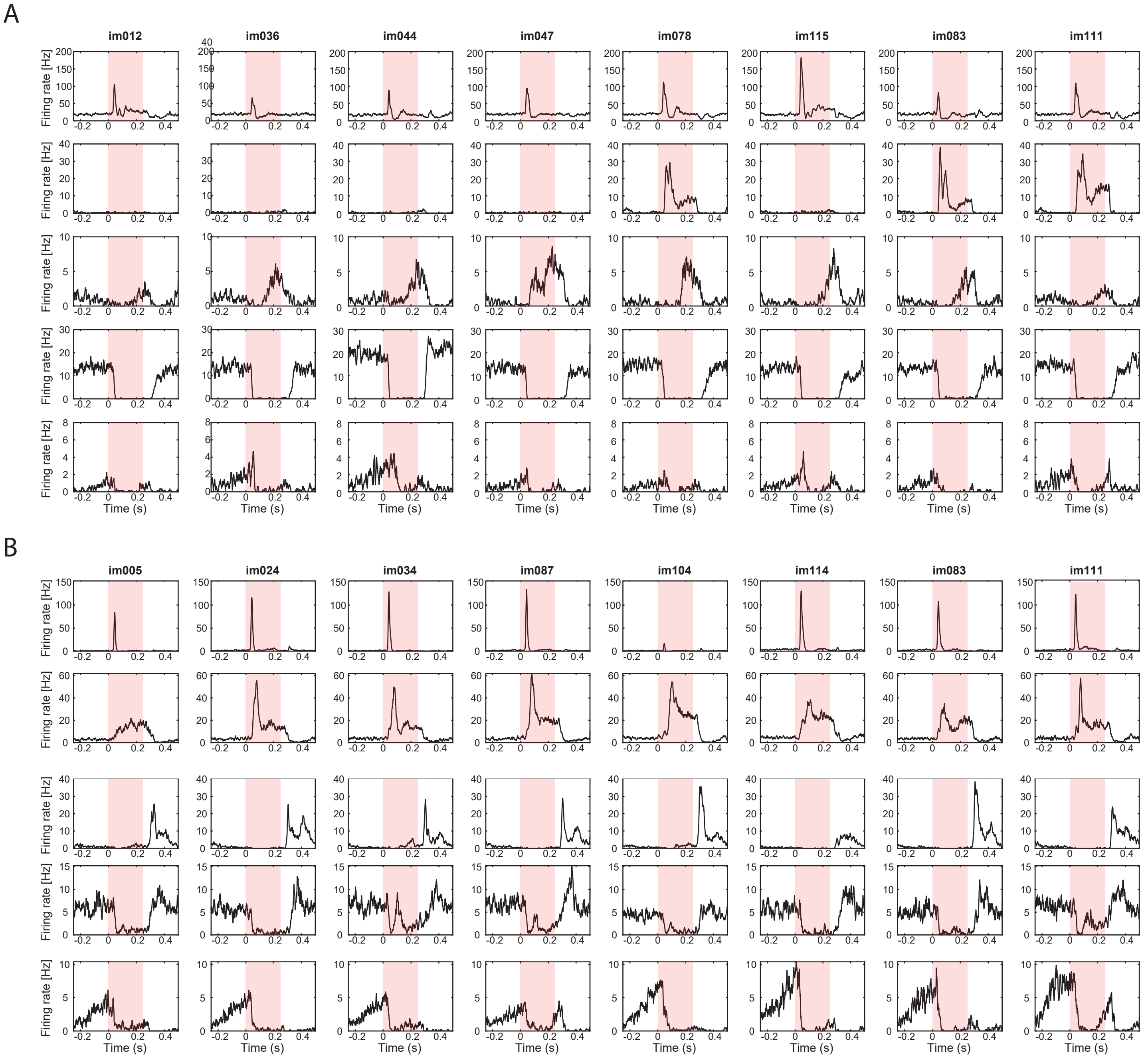

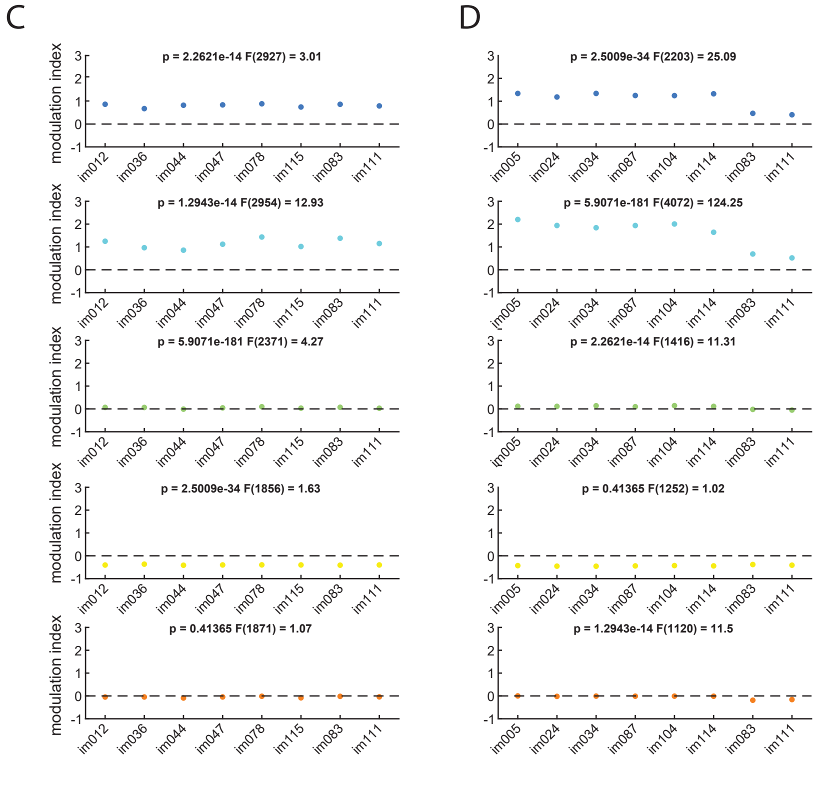

Figure S10. Clustering of visual cortex cells. A) Response of example neurons belonging to clusters 1-5 to the 8 different images presented in the task on day 1. Note that while neurons show varying degrees of sensitivity to the identity of the image, their response profile remains relatively unchanged. **B)** Same, for day 2. **C)** Modulation index (median ± 95% confidence intervals) of neurons in each cluster in response to each of the different images. 2-way ANOVA statistics and sample sizes are reported on top of each panel. Note that clusters 4-5 show diminished variability in responses to the different images. **D)** Same as C, but for day 2.

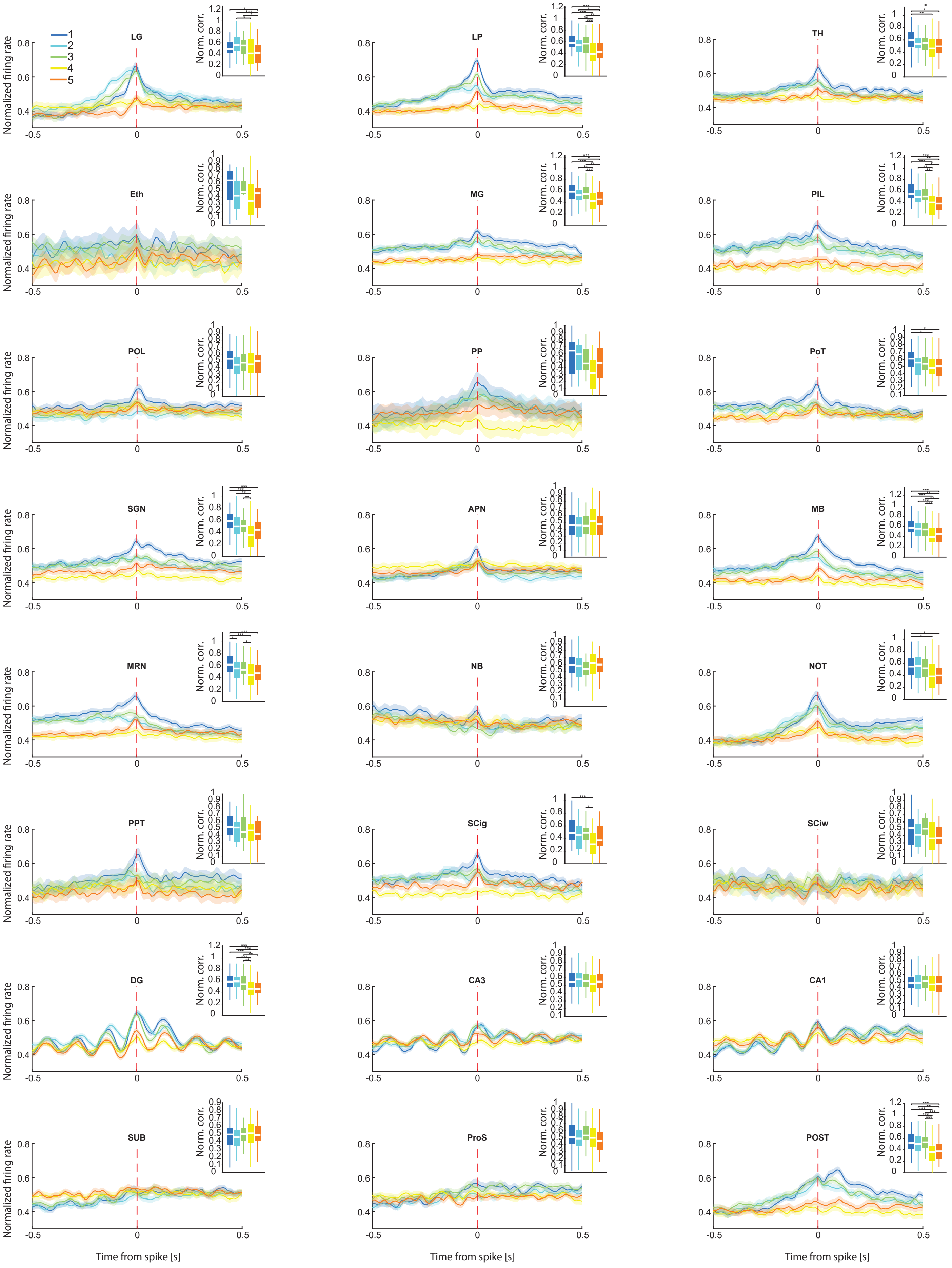

Figure S11. Correlations between visual cortex clusters and population firing in subcortical areas. Each panel depicts the normalized cross-correlograms between the different visual cortex clusters and the various subcortical areas included in the dataset. Insets, distributions of normalized correlation (area under the cross-correlograms in a window ± 20 ms around zero) for the different clusters (*p<0.05, **0<0.01, ***p<0.001; Kruskal-Wallis with Tukey-Kramer posthoc tests). Note that STIM-ON clusters lag behind thalamic nucleus involved in visual processing (LG, LP), as indicated by the negative shift of the cross-correlograms, while STIM-OFF clusters do not show a clear temporal relationship. See Fig. S2 for the numbers of units in each area.
